# The Zika Contraception Access Network: a feasibility programme to increase access to contraception in Puerto Rico during the 2016–17 Zika virus outbreak

**DOI:** 10.1016/S2468-2667(18)30001-X

**Published:** 2018-01-19

**Authors:** Eva Lathrop, Lisa Romero, Stacey Hurst, Nabal Bracero, Lauren B Zapata, Meghan T Frey, Maria I Rivera, Erin N Berry-Bibee, Margaret A Honein, Judith Monroe, Denise J Jamieson

**Affiliations:** a Department of Gynecology and Obstetrics, Emory University, Atlanta, GA, USA; b Division of Reproductive Health, National Center for Chronic Disease Prevention and Health Promotion, Centers for Disease Control and Prevention, Atlanta, GA, USA; c Division of Congenital and Developmental Disorders, National Center on Birth Defects and Developmental Disabilities, Centers for Disease Control and Prevention, Atlanta, GA, USA; d University of Puerto Rico, Área Centro Medico, San Juan, Puerto Rico; e Puerto Rico Section of the American College of Obstetricians and Gynecologists, San Juan, Puerto Rico; f Puerto Rico Obstetrics and Gynecology, San Juan, Puerto Rico; g National Foundation for the Centers for Disease Control and Prevention, Atlanta, GA, USA

## Abstract

**Background:**

Prevention of unintended pregnancy is a primary strategy to reduce adverse pregnancy and birth outcomes related to Zika virus infection. The Zika Contraception Access Network (Z-CAN) aimed to build a network of health-care providers offering client-centred contraceptive counselling and the full range of reversible contraception at no cost to women in Puerto Rico who chose to prevent pregnancy during the 2016–17 Zika virus outbreak. Here, we describe the Z-CAN programme design, implementation activities, and baseline characteristics of the first 21 124 participants.

**Methods:**

Z-CAN was developed by establishing partnerships between federal agencies, territorial health agencies, private corporations, and domestic philanthropic and non-profit organisations in the continental USA and Puerto Rico. Private donations to the National Foundation for the Centers for Disease Control and Prevention (CDCF) secured a supply of reversible contraceptive methods (including long-acting reversible contraception), made available to non-sterilised women of reproductive age at no cost through provider reimbursements and infrastructure supported by the CDCF. To build capacity in contraception service provision, doctors and clinic staff from all public health regions and nearly all municipalities in Puerto Rico were recruited into the programme. All providers completed 1 day of comprehensive training in contraception knowledge, counselling, and initiation and management, including the insertion and removal of long-acting reversible contraceptives (LARCs). Z-CAN was announced through health-care providers, word of mouth, and a health education campaign. Descriptive characteristics of programme providers and participants were recorded, and we estimated the factors associated with choosing and receiving a LARC method. As part of a Z-CAN programme monitoring plan, participants were invited to complete a patient satisfaction survey about whether they had obtained free, same-day access to their chosen contraceptive method after receiving comprehensive counselling, their perception of the quality of care they had received, and their satisfaction with their chosen method and services.

**Findings:**

Between May 4, 2016, and Aug 15, 2017, 153 providers in the Z-CAN programme provided services to 21 124 women. 20 110 (95%) women received same-day provision of a reversible contraceptive method. Whereas only 767 (4%) women had used a LARC method before Z-CAN, 14 259 (68%) chose and received a LARC method at their initial visit. Of the women who received a LARC method, 10 808 (76%) women had used no method or a least effective method of contraception (ie, condoms or withdrawal) before their Z-CAN visit. Of the 3489 women who participated in a patient satisfaction survey, 3068 (93%) of 3294 women were very satisfied with the services received, and 3216 (93%) of 3478 women reported receiving the method that they were most interested in after receiving counselling. 2382 (78%) of 3040 women rated their care as excellent or very good.

**Interpretation:**

Z-CAN was designed as a short-term response for rapid implementation of reversible contraceptive services in a complex emergency setting in Puerto Rico and has served more than 21 000 women. This model could be replicated or adapted as part of future emergency preparedness and response efforts.

**Funding:**

National Foundation for the Centers for Disease Control and Prevention.

## Introduction

Prevention of unintended pregnancy is a primary strategy to reduce adverse pregnancy and birth outcomes related to Zika virus infection.^1^, ^2^ Puerto Rico has the highest number of symptomatic Zika virus infections in the USA and US territories, including infections in women.^3^ Additionally, 65% of pregnancies in Puerto Rico are unintended, and about 138 000 of the 715 000 women aged 15–44 years in Puerto Rico are at risk for unintended pregnancy.^4^ 5–10% of the pregnancies with laboratory-confirmed Zika virus infection that were reported to the US Zika Pregnancy Registry resulted in a fetus or infant with Zika-virus-associated birth defects, and the full range of adverse development outcomes is not yet known.^5^ The threat of severe birth defects associated with Zika virus infection during pregnancy underscores the importance of contraception to prevent unintended pregnancies. However, a review of existing data and in-depth interviews with key informants early in the Zika virus outbreak in March, 2016, demonstrated that contraceptive access in Puerto Rico was limited by reduced availability of the full range of reversible methods, high out-of-pocket costs, insufficient provider reimbursement, logistical barriers that limit same-day provision, lack of patient education, and shortage of providers trained in insertion, removal, and management of long-acting reversible contraception (LARC), which includes intrauterine devices and contraceptive implants.^4^ LARC is a highly effective, safe, cost-effective, and user-friendly method of contraception that reduces unintended pregnancy and abortion.^6^, ^7^, ^8^, ^9^ In 2002–14, LARC use in the USA increased from 2·4% to 14·3% of women using contraception.^10^ However, LARC use in Puerto Rico was low before the Zika virus outbreak, with estimates indicating that less than 1% of women using contraception used a LARC method.^4^

**Research in context**

**Evidence before this study**

We searched PubMed for articles published on or before April 1, 2016, using the terms “Contraceptive Choice Project”, “Zika and family planning”, and “Zika and contraception”. The Contraceptive CHOICE Project was a prospective cohort study of 10 000 women of reproductive age in St Louis, MO, USA, who wanted to prevent pregnancy and initiate a new method of contraception. The study was designed to introduce and promote the use of long-acting reversible contraception (LARC) methods, and the results showed that 65% of participating women chose LARC methods when cost, provider, and facility barriers were removed. In a report from April 1, 2016, early in Puerto Rico's 2016–17 Zika virus outbreak, women in the country were shown to have a high unmet need for contraception, high incidence of unintended pregnancy, poor access to contraception, and the highest number of Zika infections in the USA and US territories. We did not identify any studies that described a contraception-focused programme as part of the response to the Zika virus outbreak.

**Added value of this study**

The Zika Contraception Access Network (Z-CAN) is the first to describe the large-scale implementation of a comprehensive programme to rapidly expand access to contraceptives during a major public health emergency response. The programme was implemented quickly and was able to serve more women than previous projects based on expansion of contraceptive access. Z-CAN included introduction to and education about LARC methods for both providers and patients with no previous exposure to or experience with these newer contraceptive methods.

**Implications of all the available evidence**

This large and rapidly established contraception programme could be replicated in other areas with serious and complex public health emergencies to ensure that unintended births are averted. Although this programme was developed to prevent unintended pregnancies and birth defects associated with Zika virus infection, avoiding unintended pregnancy is an important strategy for a wide variety of public health responses, particularly in view of frequent disruptions in care and services in emergency settings.

Recognising the importance of contraceptive access during the Zika virus outbreak, the National Foundation for the Centers for Disease Control and Prevention (CDCF), with technical assistance from the Centers for Disease Control and Prevention (CDC) and in collaboration with a diverse group of stakeholders and private donors, established the Zika Contraception Access Network (Z-CAN) in Puerto Rico. Z-CAN was a short-term response (from May, 2016, to September, 2017) for rapid implementation of reversible contraceptive services in a complex emergency setting. Z-CAN aimed to build a network of health-care providers trained in client-centred contraceptive counselling and same-day provision of the full range of reversible contraceptive methods (including LARC) at no cost to women who choose to delay or avoid pregnancy, and to raise awareness in women and families of contraception as a primary prevention measure to reduce adverse pregnancy and birth outcomes related to Zika virus infection. In addition to access barriers, a history of coerced sterilisation and concern for unethical testing of oral contraceptives in Puerto Rico were important considerations in programme design.^11^, ^12^

Here we describe the Z-CAN programme design and implementation activities and the baseline characteristics of the first 21 124 women served through Z-CAN.

## Methods

### Programme design and implementation

**Figure 1:**
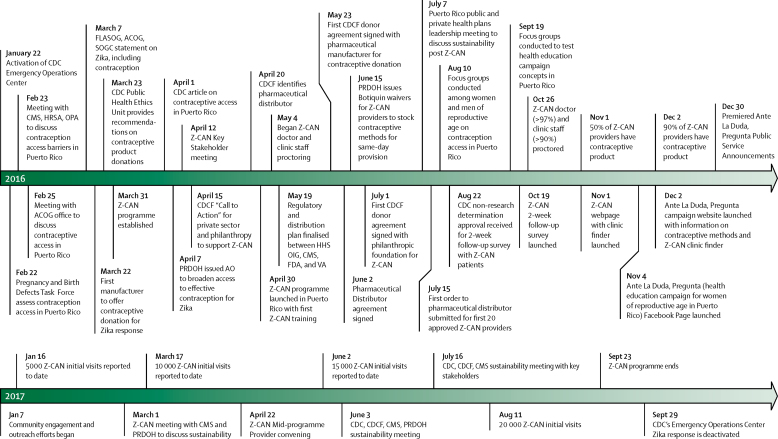
Zika Contraception Access Network (Z-CAN) major milestones, 2016–17 CDC=Centers for Disease Control and Prevention. CMS=Centers for Medicare and Medicaid Services. HRSA=Health Resources and Service Administration. OPA=Office of Population Affairs. FLASOG=Federacion Latinoamericana de Sociedades de Obstetricia y Ginecologia. ACOG=American College of Obstetricians and Gynecologists. SOGC=The Society of Obstetricians and Gynecologists. PRDOH=Puerto Rico Department of Health. AO=Administrative Order. HHS OIG=Health and Human Services Office of the Inspector General. FDA=US Food and Drug Administration. VA=Veterans Administration.

Z-CAN was designed to address gaps in contraceptive access and service provision in Puerto Rico as a preventive measure to reduce the effect of Zika virus on infants. The development of Z-CAN included several strategies to rapidly reduce access barriers to contraception in Puerto Rico's health system, strengthen infrastructure to support the Z-CAN programme, and work towards the sustainability of reversible contraceptive services after the Z-CAN programme ends (figure 1).

The development of strong partnerships was crucial in the design and implementation of Z-CAN. The programme was built with a network of partners including federal agencies, territorial health agencies, private corporations, and domestic philanthropic and non-profit organisations in the continental USA and Puerto Rico. Private donors provided product commitments to CDCF for the full range of reversible contraceptive methods (including LARC methods). CDCF established a plan for contraception procurement and distribution adherent to US Food and Drug Administration (FDA) and territorial guidelines and for private donations through CDCF-supported provider reimbursement and infrastructure costs to ensure contraception was available to women at no cost.

The gaps in contraceptive access and service provision^4^ meant that it was necessary to build provider and staff capacity in contraception knowledge, counselling, and initiation and management, including the insertion and removal of LARC. Z-CAN recruited doctors and clinic staff (nurses and clinic administrators) from all public health regions and nearly all municipalities on the island who practised in private and publicly funded clinics and who were interested in receiving training in the provision of contraception.^13^ Doctors and clinic staff were not recruited from municipalities with no community health centres, government facilities, or private practices providing women's health care. Doctors and staff were recruited through the Puerto Rico section of the American College of Obstetricians and Gynecologists, Puerto Rico Obstetrics and Gynecology, the Puerto Rico Department of Health, the Puerto Rico Primary Care Association, the Puerto Rico Health Insurance Administration, and Medicaid-managed care organisations. Before Z-CAN, none of the participating clinics routinely provided levonorgestrel-releasing intrauterine devices or contra-ceptive implants, and access to copper intrauterine devices was very limited. A 1-day comprehensive training course offered participants an overview of Zika virus (including the risk of sexual transmission and the importance of condom use for disease prevention), a tested curriculum on client-centred contraceptive counselling, didactic information about the full range of reversible contraceptives, a review of evidence-based contraceptive guidelines,^14^, ^15^ practical training in insertion and removal of intrauterine devices (providers were observed on three to five simulations),^13^ an FDA-approved etonogestrel implant training, and a overview of Z-CAN policies and procedures.

Provider reimbursement for these services was previously identified as barriers to contraception access.^4^ Through Z-CAN, private donations were used to provide a level of provider reimbursement that was commensurate with Medicaid reimbursement rates in the continental USA. This reimbursement covered client-centred contraceptive counselling for women and their partners, if desired, and method provision. If a LARC was provided, the reimbursement fee was bundled to include both insertion and removal at the time of the insertion visit to ensure that women could have their LARC devices removed when desired at no cost.

After initial training, a Z-CAN staff member and a family planning specialist proctored providers and clinic staff to ensure delivery of high-quality care. Proctoring visits consisted of: direct observation of contraceptive counselling, at least one insertion of an intrauterine device, and staff interaction with patients; review of data collection, inventory tracking, and billing procedures; and a clinic audit to ensure that supplies, space, equipment, and security were sufficient to participate in Z-CAN. If provider, staff, and clinic met all readiness criteria, they were authorised to receive contraceptive products and to begin offering Z-CAN services.

### Data collection and analysis

Women learned of Z-CAN through providers, word of mouth, and a health education campaign involving community engagement activities, Z-CAN materials, posters in health centres, a campaign website, and a Facebook page. Non-sterilised women of reproductive age were eligible to receive Z-CAN services, irrespective of age or insurance status. All Z-CAN services were provided free of charge.

At the initial Z-CAN visit, women were assigned a unique identification number. Providers and clinic staff recorded women's demographic information, reproductive and contraception histories, and their chosen contraceptive method. Data were submitted without personal identifying information to the Z-CAN programme and entered into a REDCap database hosted on a secure server.^16^

The data presented here are descriptive characteristics of programme providers and women receiving Z-CAN services. To examine factors associated with choosing and receiving a LARC method, we estimated unadjusted and adjusted prevalence ratios with 95% CI. Data were analysed using SAS-callable SUDAAN version 11.0.0 to account for clustering of patients within clinic-provider dyads.

The CDC's Public Health Ethics Committee (PHEC) provided internal consultation during the programme and project design to ensure no conflicts of interest existed and to address any ethical concerns.^17^ The Public Health Ethics Conflict of Interest Work Group, part of the CDC Zika Response Emergency Operations Center and comprised of individuals from the PHEC, reviewed the Z-CAN programme proposal during its design phase and recommended that the programme offer the full range of reversible contraceptive methods and have measures in place to prevent coercion of women.

As part of the Z-CAN programme monitoring plan, women were invited to participate in a 10 min self-administered online survey within 2 weeks of their initial visit. Z-CAN-trained clinic staff collected contact information from women who did not opt out of being contacted for future surveys. Women were invited to participate in the survey via email or text message; those without online access could complete the survey on the telephone with programme staff. The survey measured whether participants received free same-day access to the contraceptive method of their choice after receiving comprehensive counselling, patient perception of the received quality of care, and satisfaction with their chosen method and services. Perception of quality of care was measured using the validated interpersonal quality of family planning care scale,^18^ comprised of 11 items measured using a five-point Likert scale (a score of 1 means poor; a score of 5 means excellent; appendix). No personal identifiers were collected, and unique identification numbers were used to merge survey responses with initial visit data. Women were considered non-respondents if they did not complete the survey within 3 weeks after confirmed receipt of email or text message invitation and after up to three outreach attempts. Responses were collected through Survey Monkey online software, and respondents received a US$10 electronic gift card. We used SAS version 9.3 to compare baseline characteristics of survey respondents and non-respondents.

The Z-CAN programme and patient satisfaction survey were determined by CDC to be non-research public health practice activities and thus exempt from Institutional Review Board review. The programme did not obtain consent from women served by Z-CAN providers. The women received a letter at their initial visit that described the follow-up contact planned for programme monitoring purposes and were given the opportunity to opt out. Women who did not opt out were invited to participate in the patient satisfaction survey. If a woman chose to participate in the survey, she did so by consenting to the survey within the online environment.

### Role of the funding source

The philanthropic donors to CDCF had no role in programme design, data collection, data analysis, data interpretation, or writing of the report. CDC provided technical assistance in collaboration with CDCF for programme design and implementation. The cor-responding author had full access to all of the data and the final responsibility to submit for publication.

## Results

**Figure 2:**
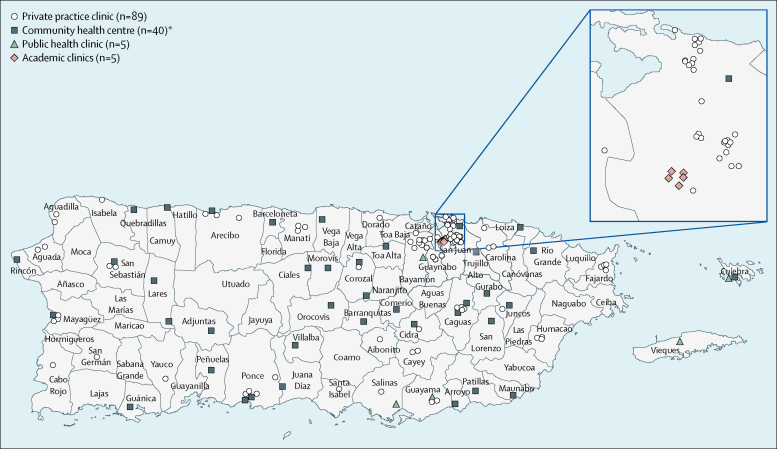
Puerto Rico Zika Contraception Access Network clinics *Includes 17 community health centres and 23 satellite clinics. Source: Zika Contraception Access Network as of Sept 23, 2017.

Training for providers took place between April 30, 2016, and Dec 6, 2016. 177 doctors, including nine resident doctors training in obstetrics and gynaecology, each participated in one of the eight Z-CAN training sessions. Of those who completed training, 153 practising doctors (141 obstetrician gynaecologists and 12 family doctors or paediatricians) agreed to participate in Z-CAN, completed proctoring visits, and received contraceptive supplies to provide Z-CAN services. The characteristics of providers are listed in table 1. 139 clinics across the island participated in the Z-CAN project (figure 2). The Z-CAN programme design, scale-up, and implementation occurred rapidly across the island, and the first Z-CAN contraception services were offered on May 4, 2016.

**Table 1:** Characteristics of Zika Contraception Access Network (Z-CAN) providers and the first 21 124 women enrolled in the Z-CAN programme, as of Aug 15, 2017

		**n/N (%)**
**Provider characteristics**		
Provider type		
	Obstetrician-gynaecologist	141/153 (92%)
	Family doctor	10/153 (7%)
	Paediatrician	2/153 (1%)
Practice type		
	Private practice	102/153 (67%)
	Community health centre*	38/153 (25%)
	Public health clinic†	3/153 (2%)
	Academic clinic‡	10/153 (7%)
**Participant characteristics**		
Age, years		
	≤20	4539/21 124 (22%)
	21–24	6057/21 124 (29%)
	25–34	7759/21 124 (37%)
	≥35	2558/21 124 (12%)
Relationship status		
	Single	8887/21 124 (42%)
	Married or partnered	11 979/21 124 (57%)
Education		
	≤12 years	7895/21 124 (37%)
	College degree	11 024/21 124 (52%)
	Graduate degree	1941/21 124 (9%)
Insurance status		
	Private or other	8813/21 124 (42%)
	Public	10 786/21 124 (51%)
	None	1111/21 124 (5%)
Previous livebirth		
	0	7762/21 124 (37%)
	≥1	12 491/21 124 (59%)
Breastfeeding at time of initial visit		
	No	17 213/21 124 (82%)
	Yes	3350/21 124 (16%)
Did not want to conceive in the next year		20 829/21 124 (95%)
Received same-day services		20 110/21 124 (95%)
Did not receive a contraceptive method at initial visit		959/21 124 (5%)
	Undecided or not ready	410/959 (43%)
	Might be pregnant	217/959 (23%)
	Desired method out of stock	97/959 (10%)
	Medical reason	83/959 (9%)
	Reason not specified	78/959 (8%)
	Did not want a contraceptive method	37/959 (4%)
	Continuing current method	26/959 (3%)
	Pregnant	11/959 (1%)

Proportions might not add up to 100% because of missing data.

* Funded by the Health Resources and Services Administration.

† Funded by the Puerto Rico Department of Public Health.

‡ Affiliated with the University of Puerto Rico.

As of Aug 15, 2017, data were available for 21 124 women who had attended an initial visit in the Z-CAN programme (table 1). The mean age of participants was 26 years (SD 6·66).

**Figure 3:**
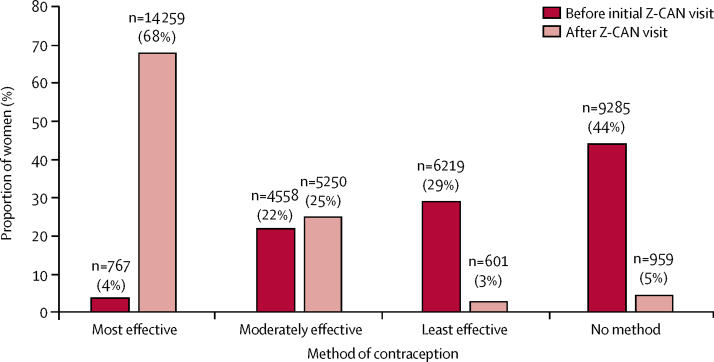
Contraceptive method use by women before and after their initial visit to a Zika Contraception Access Network (Z-CAN) provider in Puerto Rico, as of Aug 15, 2017 (N=21 124) Proportions might not add up to 100% because of missing data. Most effective contraceptive methods include intrauterine devices, implants, and partner sterilisation. Less than 1% of women using these methods will get pregnant during the first year of typical use. Moderately effective contraceptive methods include injectables, pills, patch, ring, and diaphragm. 6–12% of women using these methods will get pregnant during the first year of typical use. Least effective birth control methods include male and female condoms, withdrawal, sponge, fertility awareness methods, and spermicides. Least effective birth control methods have a failure rate of 18 or more pregnancies per 100 women who use these methods each year. The Centres for Disease Control and Prevention have produced an overview of the effectiveness of family planning methods. Methods provided by Z-CAN included intrauterine devices, implants, injectables, pills, patch, ring, and male condoms.

The distribution of contraception methods used by women before and after joining the Z-CAN programme is shown in figure 3. Before their initial Z-CAN visit, most women used either no method or one of the least effective contraceptive methods (condoms, sponge, withdrawal, spermicide, or fertility awareness methods), and only a small proportion of women used one of the most effective methods (male sterilisation, intrauterine device, or implant; figure 3). At their visit, more than 14 259 (68%) women chose and received a LARC method and 5250 (25%) women chose oral contraceptive pills or other moderately effective hormonal contraception (eg, depot medroxyprogesterone acetate injection). Of the 959 (5%) women who did not receive a contraceptive method, the most common reasons were being undecided on method preference or not ready to receive the method that day, pregnancy could not be ruled out, or the desired method was not in stock (table 1). Of the 14 259 women who chose and received a LARC method, 7167 (50%) women received a levonorgestrel-releasing intrauterine device, 5031 (35%) women received an etonogestrel implant, and 2061 (14%) women received a copper intrauterine device. Women were more likely to choose and receive a LARC method if they had a college degree, had no insurance, had at least one livebirth, used a most effective contraceptive method before Z-CAN, and saw a Z-CAN provider in private practice or a public health or academic clinic, after adjustment for all other characteristics (table 2). Women aged 25 years or more and women using a moderately effective contraceptive method before Z-CAN were less likely to choose and receive a LARC method. Results were similar when the analysis was restricted to women who received a contraceptive method at their initial visit.

**Table 2:** Factors associated with choosing and receiving a LARC method among the first 21 124 women enrolled in the Zika Contraception Access Network (Z-CAN) programme, as of Aug 15, 2017

	**LARC (n=14 259)**	**Other contraceptive method (n=6810)**	**Unadjusted prevalence ratio, 95% CI**	Adjusted prevalence ratio, 95% CI*
**Age, years**				
≤20	2930/14 125 (21%)	1594/6734 (24%)	Referent	Referent
21–24	4176/14 125 (30%)	1868/6734 (28%)	1·07, 1·03–1·10†	1·00, 0·97–1·03
25–34	5305/14 125 (38%)	2435/6734 (36%)	1·06, 1·02–1·10†	0·93, 0·90–0·97†
≥35	1714/14 125 (12%)	837/6734 (12%)	1·04, 0·98–1·10	0·85, 0·80–0·92†
**Relationship status**				
Single	5717/14 106 (41%)	3148/6709 (47%)	Referent	Referent
Married or partnered	8389/14 106 (60%)	3561/6709 (53%)	1·09, 1·04–1·14†	0·99, 0·95–1·04
**Education**				
≤12 years	5258/14 094 (37%)	2617/6712 (39%)	Referent	Referent
College degree	7585/14 094 (54%)	3411/6712 (51%)	1·03, 1·00–1·07	1·04, 1·01–1·08†
Graduate degree	1251/14 094 (9%)	684/6712 (10%)	0·97, 0·91–1·03	1·02, 0·96–1·08
**Insurance status**				
Private or other	5827/13 970 (42%)	2968/6689 (44%)	Referent	Referent
Public	7326/13 970 (52%)	3429/6689 (51%)	1·03, 0·97–1·09	0·97, 0·91–1·02
None	817/13 970 (6%)	292/6689 (4%)	1·11, 1·05–1·18†	1·11, 1·05–1·17†
**Previous livebirth**				
0	4301/13 688 (31%)	3431/6511 (53%)	Referent	Referent
1 or more	9387/13 688 (69%)	3080/6511 (47%)	1·35, 1·27–1·44†	1·40, 1·31–1·48†
**Currently breastfeeding**				
No	11 271/13 884 (81%)	5892/6626 (89%)	Referent	Referent
Yes	2613/13 884 (19%)	734/6626 (11%)	1·19, 1·14–1·24†	1·03, 0·99–1·08
**Effectiveness of contraceptive method used before Z-CAN**‡				
None	6357/14 097 (45%)	2909/6683 (44%)	Referent	Referent
Least	4451/14 097 (32%)	1757/6683 (26%)	1·05, 0·98–1·11	1·05, 0·99–1·11
Moderately	2666/14 097 (19%)	1874/6683 (28%)	0·86, 0·82–0·89†	0·90, 0·86–0·94†
Most	623/14 097 (4%)	143/6683 (2%)	1·19, 1·12–1·25†	1·13, 1·06–1·21†
**Clinic type**				
Community health clinic	2154/14 259 (15%)	1521/6810 (22%)	Referent	Referent
Private practice or other	12 105/14 259 (85%)	5289/6810 (78%)	1·19, 1·06–1·33†	1·19, 1·07–1·33†

Data are n/N (%) unless indicated otherwise. LARC=long-acting reversible contraceptive.

* Each characteristic in the table was adjusted for all other characteristics.

† 95% CI does not include 1.

‡ Least effective contraceptive methods include condoms for men and women, withdrawal, sponge, fertility awareness methods, and spermicides. Moderately effective contraceptive methods include injectables, pills, patch, ring, and diaphragm. Most effective contraceptive methods include intrauterine devices, implants, and partner sterilisation. Sterilised women were not eligible for Z-CAN services.

The satisfaction survey began on Oct 28, 2016. By July 21, 2017, 9829 women had received invitations to complete the patient satisfaction survey, and 3489 (36%) women had responded (2482 women responded by email invitation, 1006 women responded by text message invitation, and one woman responded by phone administration). We were able to link initial visit data to survey data for 3439 (99%) respondents. Respondents differed from non-respondents with respect to age, insurance status, and type of method received; compared with non-respondents, respondents overall were slightly older, had private insurance, and chose a more effective method during their visit. 3489 women participated in the patient satisfaction survey, but not all women completed every question of the survey. 3068 (93%) of the 3294 women who answered the question about their satisfaction with services were very satisfied, 203 (6%) women were somewhat satisfied, and 23 (1%) women were not satisfied. 3216 (93%) of the 3478 women who answered the question about receiving the method they were most interested in after receiving counselling did receive the method they were most interested in. Of the 3040 women who completed every item on the 11-item interpersonal quality of family planning care scale, 2382 (78%) respondents rated their care as excellent or very good on all 11 items. Results from individual items measuring quality of care are summarised in the appendix.

## Discussion

In Puerto Rico, the combination of a high incidence of Zika virus infection, a high incidence of unintended pregnancy, and low use of highly effective contraception necessitated programmatic efforts to improve con-traceptive access as a primary prevention strategy to reduce adverse pregnancy and birth outcomes related to Zika virus infection. The Z-CAN programme shows the feasibility of implementing a programme to increase access to the full range of reversible contraception, including LARC methods, within a complex public health response. Z-CAN also shows that it is possible to build capacity quickly with standardised and targeted training sessions and limited mentoring of committed providers and to provide high-quality, comprehensive contraceptive services in an emergency response.

Contraception has an important role in the Zika response because Zika virus infection during pregnancy increases the risk for microcephaly and other severe birth defects.^2^ Contraception could be a key response strategy in other public health emergencies in which prenatal exposures pose a severe risk to pregnant women and their infants.^19^ Guidance for rapid reproductive health assessment and programme implementation in emergency settings is available, but existing tools position contraception services as post-emergency activities rather than services to be implemented in the emergency phase.^20^ Z-CAN shows that with concerted effort, commitment, dedicated resources, and recognition of the benefits of giving women the option to prevent pregnancy during a time of crisis, it is possible to prioritise and implement effective contraceptive provision early in an emergency response.

Contraceptive use and provision in Puerto Rico before the Z-CAN programme was limited by policy, financial, and logistical barriers.^4^, ^21^ Most of the 21 124 women seen by the Z-CAN programmme chose and received a LARC method, and most of these women were not using an effective method of contraception before Z-CAN; these findings suggest that when barriers to access are removed (eg, cost, limited service points, and lack of providers), most women who wanted to prevent pregnancy during the Zika virus outbreak chose a highly effective method of contraception. The choice of a LARC method was more likely in women who had previously given birth than in nulliparous women. Intrauterine devices are generally safe for all women, including nulliparous women.^14^ Providers might have misconceptions about the safety of intrauterine devices in nulliparous women, which have been shown to be associated with infrequent provision,^22^ emphasising the opportunity for providers to include LARC methods in counselling and eligibility determinations for all women seeking contraception. Although use of LARC methods by women using contraception in the USA is low (14%),^10^ our findings are consistent with those from other demonstration projects^9^, ^23^ that removed barriers to LARC access such as cost, provider availability, geographic access, and comprehensive contraception counselling. Women who chose a short-acting method were given up to 6 months advanced supply. Women who perceived a return visit to receive additional contraceptive supplies as a barrier might have inadvertently been incentivised to choose a LARC method. However, results from the patient satisfaction survey suggested that most women left their initial Z-CAN visit with the method they were most interested in receiving. In the context of the Zika virus outbreak, improved access to contraception has the potential to decrease unintended pregnancies and the number of adverse pregnancy and birth outcomes related to Zika virus infection.^1^, ^4^, ^24^

On the basis of results from multiple large-scale programmes and research studies to reduce barriers to contraceptive access, we anticipated that Z-CAN services would lead to an increase in LARC use. Because of their many advantages, including high effectiveness, safety, reversibility, user ease, high user satisfaction, and cost-effectiveness, LARC methods are crucial in public health efforts to decrease unintended pregnancies. However, issues of perceived or actual provider coercion of women to choose LARC methods (or refuse LARC removals), particularly based on age, race, and class, have been reported.^25^, ^26^ The historical context of unethical contraceptive practices and research in Puerto Rico and concerns for reproductive coercion with LARC provision were important considerations in programme design. An important element of the Z-CAN training and proctoring for all providers and clinic staff was to develop competency in delivering high-quality, patient-centred contraceptive counselling that facilitated autonomous decision making.^13^ Respondents to the satisfaction survey indicated high satisfaction with Z-CAN services, and nearly all women received the method they were most interested in after counselling, suggesting that participants received high-quality and patient-centred services through Z-CAN. The Z-CAN programme evaluation will include additional follow-up surveys of women participating in the programme to further assess quality of and satisfaction with Z-CAN services.

Through partnership and collaboration with a diverse group of stakeholders, Z-CAN reduced barriers to contraception as part of the public health response to the Zika virus outbreak and expanded the capacity of Puerto Rico's health-care system to integrate same-day access to contraceptive services into normal clinic practice. Z-CAN efforts to build sustainability with key stakeholders include building the capacity of a broad network of providers who can provide access to contraception, raising awareness in women of reproductive age in Puerto Rico about the availability of contraceptive methods, expanding the number of contraceptive service access sites, eliminating prior authorisation requirements and cost-sharing in health insurance plans, and discussing continued availability of LARC methods in Puerto Rico through pricing negotiations and development of a sustainable supply chain with manufacturers. Although the total cost to implement, sustain, or replicate the Z-CAN programme is difficult to calculate, the most expensive aspects of the programme were provision of the contraceptive methods (almost all of which were donated in the case of Z-CAN) and provider reimbursement for services. Different contexts will have different cost challenges, but the financing requirements of these crucial aspects might be substantial and should be considered in programme design and sustainability planning. Successful sustainability will be achieved if the elimination of the most pressing barriers addressed by Z-CAN is maintained.

This programme has several strengths. To our knowledge, Z-CAN is the first contraception access programme developed as a primary prevention strategy to mitigate the effect of a Zika virus outbreak, and it is the first contraception access programme as a primary intervention to prevent adverse pregnancy and birth outcomes in the context of a public health emergency response. The Z-CAN programme contains important elements of both rapid programme design and implementation and sustainability planning and can be adapted to other settings in which improving contraceptive access could enhance the response to an emergency. The strong partnerships between programme teams and stakeholders in Puerto Rico and the high demand for contraceptive services also strengthened the programme.

The Z-CAN programme and this study also have several limitations. Although Z-CAN had broad coverage across the island, the programme was not able to provide services in municipalities without health-care infrastructure, so some women had to travel outside their municipality to access care. Because of the rapid design and implementation of Z-CAN and the specific threat of Zika virus to maternal and child health, our results are not readily generalisable to non-emergency situations. The response rate to the patient satisfaction survey was low, and the results of the survey might not be generalisable to all women who received Z-CAN services. The programme was implemented to serve women throughout the risk period for Zika virus transmission, while working towards sustainability of high-quality and accessible contraceptive services. Although the design and implementation phases were relatively fast, rate-limiting steps (eg, design of a procurement and distribution system for donated contraceptive methods) slowed the delivery of services in the early phases of the programme. In view of the challenges of procurement and payment of LARC methods, reaching a level of sustainability of contraceptive services that closely mimics Z-CAN will probably be difficult.^21^

Z-CAN was designed as a short-term response for rapid implementation of contraceptive services in a complex emergency setting. Z-CAN has established an extensive network of providers in Puerto Rico and has served more than 21 000 women seeking to prevent pregnancy during the risk period for Zika virus infection. The programme might have prevented unintended pregnancies and birth defects related to Zika virus infections during the outbreak. Mosquito-borne transmission of Zika virus has reached 95 countries worldwide and all but two countries in the Latin America Caribbean Region.^27^ On the basis of these preliminary results, Z-CAN is a model programme that could be replicated or adapted in these settings as part of emergency preparedness and response efforts. Additionally, Z-CAN's design and implementation could be refined and adapted in other non-emergent settings, in which increased access to contraception could improve health outcomes.

For the **effectiveness of family planning methods** see https://www.cdc.gov/reproductivehealth/unintendedpregnancy/pdf/contraceptive_methods_508.pdf
