# Supplementary material for: The Zika Contraception Access Network: a feasibility programme to increase access to contraception in Puerto Rico during the 2016–17 Zika virus outbreak
Source: Lancet Public Health. 2018 Jan 19;3(2):e91–9. doi: 10.1016/S2468-2667(18)30001-X (PMC5846878; doi:10.1016/S2468-2667(18)30001-X)
Supplement: Supplementary appendix [file mmc1.pdf]

THE LANCET
Public Health

Supplementary appendix

This appendix formed part of the original submission and has been peer reviewed. We post it as supplied by the authors.

Supplement to: Lathrop E, Romero L, Hurst S, et al. The Zika Contraception Access Network: a feasibility programme to increase access to contraception in Puerto Rico during the 2016–17 Zika virus outbreak. *Lancet Public Health* 2018; published online Jan 18. http://dx.doi.org/10.1016/S2468-2667(18)30001-X.

Appendix: Survey items used to measure patient perception of quality of care in the Z-CAN patient satisfaction survey[a] and findings

On a scale of 1 to 5, with *1 meaning poor and 5 meaning excellent*, please rate your experience with your Z-CAN provider(s) (doctor, nurse, and/or counselor) that you saw according to the following qualities:	1=Poor	2=Fair	3=Good	4=Very good	5=Excellent
Respecting me as a person.	9/3428 (0·3)	21/3428 (0·6)	63/3428 (1·8)	226/3428 (6·6)	2719/3428 (79·3)
Showing care and compassion.	18/3428 (0·5)	22/3428 (0·6)	93/3428 (2·7)	260/3428 (7·6)	2641/3428 (77·0)
Letting me say what mattered to me about my birth control method.	12/3428 (0·4)	27/3428 (0·8)	108/3428 (3·2)	259/3428 (7·6)	2627/3428 (76·6)
Giving me an opportunity to ask questions.	19/3428 (0·6)	25/3428 (0·7)	94/3428 (2·7)	208/3428 (6·1)	2690/3428 (78·5)
Taking my preferences about my birth control seriously.	22/3428 (0·6)	30/3428 (0·9)	90/3428 (2·6)	251/3428 (7·3)	2642/3428 (77·1)
Considering my personal situation when advising me about birth control.	32/3428 (0·9)	37/3428 (1·1)	116/3428 (3·4)	255/3428 (7·4)	2588/3428 (75·5)
Working out a plan for my birth control with me.	138/3428 (4·0)	102/3428 (3·0)	248/3428 (7·2)	368/3428 (10·7)	2171/3428 (63·3)
Giving me enough information to make the best decision about my birth control method.	28/3428 (0·8)	63/3428 (1·8)	117/3428 (3·4)	248/3428 (7·2)	2572/3428 (75·0)
Telling me how to take or use my birth control method most effectively.	24/3428 (0·7)	35/3428 (1·0)	126/3428 (3·7)	241/3428 (7·0)	2605/3428 (76·0)
Telling me the risks and benefits of the birth control method I chose.	64/3428 (1·9)	78/3428 (2·3)	135/3428 (3·9)	297/3428 (8·7)	2454/3428 (71·6)
Answering all of my questions.	17/3428 (0·5)	42/3428 (1·2)	108/3428 (3·2)	232/3428 (6·8)	2629/3428 (76·7)

[a] Adapted from Dehlendorf C, Henderson JT, Vittinghoff E, Grumbach K, Levy K, Schmittdiel J, et al. Association of the quality of interpersonal care during family planning counseling with contraceptive use. *Am J Obstet Gynecol.* 2016;215(1):78.e1–9.

Note: Proportions may not add to 100% due to missing data.
